# Simultaneous assessment of cytotoxic T lymphocyte responses against multiple viral infections by combined usage of optimal epitope matrices, anti- CD3 mAb T-cell expansion and "RecycleSpot"

**DOI:** 10.1186/1479-5876-3-20

**Published:** 2005-05-11

**Authors:** Florian K Bihl, Elisabetta Loggi, John V Chisholm, Hannah S Hewitt, Leah M Henry, Caitlyn Linde, Todd J Suscovich, Johnson T Wong, Nicole Frahm, Pietro Andreone, Christian Brander

**Affiliations:** 1 Partners AIDS Research Center, Massachusetts General Hospital, Harvard Medical School, Boston, USA; 2 Department of Pathology, Massachusetts General Hospital, Harvard Medical School, Boston, USA; 3 Dipartimento di Cardioangiologia ed Epatologia, Ospedale S. Orsola-Malpighi, Università degli Studi di Bologna, Italy

**Keywords:** Cytotoxic T Cells, HIV, EBV, CMV, HCV, HBV, CTL, epitope, peptide, cell expansion, anti-CD3, ELISpot, peptide matrix

## Abstract

The assessment of cellular anti-viral immunity is often hampered by the limited availability of adequate samples, especially when attempting simultaneous, high-resolution determination of T cell responses against multiple viral infections. Thus, the development of assay systems, which optimize cell usage, while still allowing for the detailed determination of breadth and magnitude of virus-specific cytotoxic T lymphocyte (CTL) responses, is urgently needed. This study provides an up-to-date listing of currently known, well-defined viral CTL epitopes for HIV, EBV, CMV, HCV and HBV and describes an approach that overcomes some of the above limitations through the use of peptide matrices of optimally defined viral CTL epitopes in combination with anti-CD3 in vitro T cell expansion and re-use of cells from negative ELISpot wells. The data show that, when compared to direct ex vivo cell preparations, antigen-unspecific in vitro T cell expansion maintains the breadth of detectable T cell responses and demonstrates that harvesting cells from negative ELISpot wells for re-use in subsequent ELISpot assays (RecycleSpot), further maximized the use of available cells. Furthermore when combining T cell expansion and RecycleSpot with the use of rationally designed peptide matrices, antiviral immunity against more than 400 different CTL epitopes from five different viruses can be reproducibly assessed from samples of less than 10 milliliters of blood without compromising information on the breadth and magnitude of these responses. Together, these data support an approach that facilitates the assessment of cellular immunity against multiple viral co-infections in settings where sample availability is severely limited.

## Introduction

Cell-mediated immunity is considered critical for the prevention and control of many viral infections [1-6]. The approaches developed to detect these responses in vitro have evolved over the years and have provided quantitative and qualitative information on virus-specific T cells for a number of viral infections. These assays include, besides others, lymphoproliferative assays using ^3^H-thymidine incorporation or CFSE staining, limiting dilution precursor-frequency assays for the enumeration of CTL precursor frequencies, intracellular cytokine staining (ICS) and enzyme-linked immunospot (ELISpot) assays [7-10]. Although these assays differ in their minimal cell requirements, the detailed, simultaneous analysis of anti-viral immunity against multiple viral infections is often limited by cell availability, regardless of the assay employed.

The ELISpot assay has become widely used for rapidly assessing cellular immune responses to extensive numbers of antigens while using relatively few cells. A number of studies have also employed peptide matrix approaches, where every antigenic peptide is tested in two peptide pools, so that responses to reactive pools sharing a specific peptide can help to identify the targeted peptide [9,11]. This has reduced the required cell numbers significantly, so that for instance HIV-specific responses can generally be comprehensively assessed using less than 15 × l0^6 ^cells [9]. However, despite such advances, the simultaneous enumeration of virus-specific immunity to multiple viral infections still exceeds the required sample size that can routinely be obtained. Sample size may not be of great concern when assessing CTL mediated immune responses against single, small genome viruses such as HIV and HCV, which can be tested in a comprehensive manner using overlapping peptide sets spanning the entire expressed viral genome [9,12]. Nevertheless, such comprehensive approaches are not feasible for larger viruses, such as DNA-based herpesviruses like EBV, CMV and KSHV [4,13]. Instead, immune analyses need either to be restricted to a selected number of specific viral proteins, or to the use of previously defined, optimal CTL epitopes. Responses against such optimally defined epitopes can account for a significant part of the total virus-specific immune responses, especially when they represent immunodominant epitopes covering the most immunogenic proteins of specific viral genomes. For well-studied viruses such as HIV, HCV, EBV and CMV, large sets of such optimally defined CTL epitopes, restricted by common HLA alleles, have been described in the past [14-17], and provide a valuable alternative to measure pathogen-specific CTL responses without the need to synthesize comprehensive peptide sets spanning the entire viral genomes.

The present study describes an algorithm by which matrices of optimally defined CTL epitopes derived from five different human viral infections are used in the same ELISpot assay. As not all wells of the ELISpot plate contain antigens to which the tested PBMCs will respond, there are consistently some wells with cells that have not been stimulated during this first assay. Theoretically, these cells could be recovered from the ELISpot plate before developing it and re-used in subsequent analyses. Indeed, others have suggested the use of "recycled" cells for DNA isolation[18], however, to our knowledge, no data exist on re-using these cells in functional assays. Since the peptide matrix approach is ideally followed by the subsequent confirmation of single targeted peptides present in two corresponding peptide pools, recycled cells from unstimulated ELISpot wells could be used for these assays. Although this second step could be achieved using in vitro expanded cells, for instance by anti-CD3 monoclonal antibody (mAb) stimulation, expanded cells may lose some of the responses compared directly to cells tested ex vivo [19,20]. In addition, the absolute and relative magnitude of responses may be distorted during cell expansion and assays can only be run after prolonged in vitro culture[21,22] Therefore, as long as functionality of recycled cells in secondary assays can be ensured, they may provide a simple way to complete initial ELISpot screenings, yielding reliable information on the magnitude of specific CTL responses. The feasibility of this approach was tested and it was shown that combined use of optimal epitope matrices, in vitro T cell expansion and RecycleSpot can provide relevant immune data on multiple viral infections even when cell availability is severely limited.

## Materials and methods

### Isolation of fresh PMBCs from whole blood

Whole blood was collected using Citrate Vacutainer tubes (BD, Franklin Lakes, NJ) and peripheral blood mononuclear cells (PBMC) were isolated by Histopaque (Histopaque^® ^1077, Sigma, St. Louis, MO) density centrifugation as described [9]. Fresh PBMC were either used directly after isolation, after in vitro expansion or after freezing and thawing with and without subsequent in vitro expansion. For in vitro use, cells were re-suspended in R10 medium (RPMI 1640 containg 10% heat inactivated FCS (both Sigma), 2 mM L-glutamine, 50 U/ml penicillin, 50 μg/ml streptomucin and 10 mM HEPES (all Mediatech, Hemdon, VA)) at a concentration of 1 × 10^6 ^cells/ml. Cells were thawed using R10 medium containing 50 U/ml DNAse (Deoxyribnuclease I, RNase-free, Sigma), washed twice in the same medium, re-suspended in R10 and incubated at 37°C with 5% CO_2 _for 3–4 hours before they were counted and re-suspended in R10 at 1 × 10^6 ^cells/ml. The thawed cells were then either used directly in ELISpot assays or expanded.

For in vitro expansion, 1 to 5 × 10^6 ^PBMC were added to 25 ml culture flasks in 10 ml R10 supplemented with 1 μl of the anti-CD3 specific monoclonal antibody (mAb) 12F6 [23]. Cells were fed twice a week using R10 supplemented with 50 U/ml of recombinant Interleukin 2 (IL-2) for 2 weeks. Before use in ELISpot assays, cells were washed twice in R10 medium and incubated overnight at 37°C with 5% CO_2 _in the absence of IL-2. This overnight starving step was necessary to eliminate background in the subseqnet ELISpot assay, which was, in our hands, not an issue, regardless of how long the *in vitro *culture had been maintained.

### Design of Optimal Peptide Matrix

A total of 416 optimal epitopes from five different viruses were assembled in 98 different peptide pools and used in 5 peptide matrices each containing peptides from a single virus. The number of pools and total number of peptides contained in each virus-specific peptide matrix are summarized in Table 1. Each peptide was present at a final concentration of 200 μg/ml in the peptide pools. Detailed lists of all optimal epitopes included in this study, along with their sequence and HLA restriction, are given in Tables 2 through 6.

**Table 1:** Virus specific peptide matrix design using previously defined HLA class I restricted CTL epitopes

**Virus**	**Optimal epitopes**	**No. of peptide pools**	**Max. no. of peptides per pools**
**HIV**	173	29	14
**EBV**	91	23	12
**CMV**	38	13	7
**HCV**	77	19	11
**HBV**	37	14	8

**Table 2:** Optimal HIV-derived HLA class I restricted CTL epitopes

Protein	HLA Restriction	Sequence	Position
gp120	A02	RGPGRAFVTI	311–320
gp120	A03	TVYYGVPVWK	37–46
gp120	A11l	SVITQACPK	199–207
gp120	A24	LFCASDAKAY	53–62
gp120	A29	SFEPIPIHY	209–217
gp120	A30	HIGPGRAFY	310–318
gp120	A32	RIKQIINMW	419–427
gp120	B07	RPNNNTRKSI	303–312
gp120	B08	RVKEKYQHL	2–10
gp120	B1516/Cw04	SFNCGGEFF	379–387
gp120	B3801	MHEDIISLW	104–112
gp120	B35	VPVWKEATTTL	42–52
gp120	B35	DPNPQEVVL	77–85
gp120	B44	AENLWVTVY	30–38
gp120	B51	LPCRIKQII	416–424
gp120	B55	VPVWKEATTT	42–51
gp120	A33	VFAVLSIVNR	187–196
gp120	A33	EVAQRAYR	320–327
gp41	A01	RRGWEVLKY	787–795
gp41	A02	SLLNATDIAV	818–827
gp41	A0205	RIRQGLERA	335–343
gp41	A03/A30	RLRDLLLIVTR	775–785
gp41	A23/A24	RYLKDQQLL	591–598
gp41	A30	IVNRNRQGY	704–712
gp41	A30	KYCWNLLQY	794–802
gp41	A6802	IVTRIVELL	782–790
gp41	B07	IPRRIRQGL	843–851
gp41	B08	YLKDQQLL	591–598
gp41	B08	RQGLERALL	848–856
gp41	B14	ERYLKDQQL	589–597
gp41	B2705	GRRGWEALKY	791–799
gp41	B35	TAVPWNASW	611–619
gp41	B4001	QELKNSAVSL	810–819
gp41	Cw3/Cw15	RAIEAQQHL	46–54
p17	A02	SLYNTVATL	77–85
p17	A03	KIRLRPGGK	18–26
p17	A03	RLRPGGKKK	20–28
p17	A03	RLRPGGKKKY	20–29
p17	A11	TLYCVHQRI	84–92
p17	A24	KYKLKHIVW	28–36
p17	A30	RSLYNTVATLY	74–86
p17	B08	GGKKKYKL	24–31
p17	B08	ELRSLYNTV	74–82
p17	B2705	IRLRPGGKK	19–27
p17	B35	WASRELERF	36–44
p17	B35	NSSKVSQNY	124–132
p17	B4001	IEIKDTKEAL	92–101
p17	B4002	GELDRWEKI	11–19
p24	A0207	YVDRFYKTL	164–172
p24	A11	ACQGVGGPGHK	349–359
p24	A24/B44	RDYVDRFFKTL	296–306
p24	A25	QAISPRTLNAW	145–155
p24	B07	SPRTLNAWV	148–156
p24	B07/B42/B81/Cw8	TPQDLNTML	48–56
p24	B07	GPGHKARVL	223–231
p24	B07	HPVHAGPIA	84–92
p24	B08	EIYKRWII	260–267
p24	B08	DCKTILKAL	329–337
p24	B14	DRFYKTLRA	298–306
p24	B1501	GLNKIVRMY	267–277
p24	B18	FRDYVDRFYK	293–302
p24	B2703	RRWIQLGLQK	260–269
p24	B2705	KRWIILGLNK	265–274
p24	B35	NPVPVGNIY	245–253
p24	B35	PPIPVGDIY	254–262
p24	B39	GHQAAMQML	193–201
p24	B4001	SEGATPQDL	176–184
p24	B4002	KETINEEAA	70–78
p24	B4002	AEWDRVHPV	78–86
p24	B44	AEQASQDVKNW	174–184
p24	B44	EEKAFSPEV	28–36
p24	B52	RMYSPTSI	143–150
p24	B53	TPYDINQML	48–56
p24	B53/B57	QASQEVKNW	176–184
p24	B57	ISPRTLNAW	15–23
p24	B57	KAFSPEVIPMF	30–40
p24	B57	TSTLQEQIGW	108–118
p24	B57	KAFSPEVI	30–37
p24	B58	TSTLQEQIGW	108–117
p24	B58	TSTVEEQIQW	108–117
p24	Cw0I	VIPMFSAL	36–43
p24	A25	ETINEEAAEW	71–80
p24	A26	EVIPMFSAL	35–43
p15	A02	FLGKIWPSYK	1–10
p15	B14	CRAPRKKGC	42–50
p15	B4001	KELYPLTSL	33–41
p15	B4002	TERQANFL	64–71
Protease	A6802/A74	ITLWQRPLV	3–11
Protease	A6802	DTVLEEMNL	30–38
Integrase	A30	KIQNFRVYY	219–227
Integrase	A03/A11	AVFIHNFKRK	179–188
Integrase	B1503	RKAKIIRDY	263–271
Integrase	B42	VPRRKAKII	260–268
Integrase	B57	KTAVQMAVF	173–181
RT	A26	ETKLGKAGY	604–612
RT	A02	ALVEICTEM	33–41
RT	A02	VIYQYMDDL	179–187
RT	A02	ILKEPVHGV	309–317
RT	A03	ALVEICTEMEK	33–43
RT	A03	GIPHPAGLK	93–101
RT	A03/A1 1	AIFQSSMTK	158–166
RT	A03	QIYPGIKVR	269–277
RT	A03	KLVDFRELNK	73–82
RT	A03	RMRGAHTNDVK	356–366
RT	A11	IYQEPFKNLK	341–350
RT	A11	QIIEQLIKK	80–88
RT	B51	TAFTIPSI	128–135
RT	B57	IVLPEKDSW	244–252
RT	B58	IAMESIVIW	375–383
RT	B81	LFLDGIDKA	715–723
RT	B1503	VTDSQYALGI	651–660
RT	A30	KQNPDIVIY	173–181
RT	A30	KLNWASQIY	263–271
RT	A30	RMRGAHTNDV	356–365
RT	A32	PIQKETWETW	392–401
RT	B08	GPKVKQWPL	18–26
RT	B1501	LVGKLNWASQIY	260–271
RT	B1501	IKLEPVHGVY	309–318
RT	B35	TVLDVGDAY	107–115
RT	B35	VPLDEDFRKY	118–127
RT	B35	NPDIVIYQY	175–183
RT	B35	HPDIVIYQY	175–183
RT	B4001	IEELRQHLL	202–210
RT	B42	YPGIKVRQL	271–279
RT	B51	EKEGKISKI	42–50
Vpr	A02	AIIRILQQL	59–67
Vpr	B07/B81	FPRIWLHGL	34–42
Vpr	B51	EAVRHFPRI	29–37
Vpr	B57	AVRHFPRIW	30–38
Tat	A6801	ITKGLGISYGR	39–49
Tat	B1503	FQTKGLGISY	38–47
Tat	B53	EPVDPRLEPW	2–11
Tat	Cw12	CCFHCQVC	30–37
Vif	A03	RIRTWKSLVK	17–26
Vif	A03	HMYISKKAK	28–36
Vif	A03	KTKPPLPSVKK	158–168
Vif	B07	HPRVSSEVHI	48–57
Vif	B18	LADQLIHLHY	102–111
Vif	B57	ISKKAKGWF	31–39
Nef	A02	PLTFGWCYKL	136–145
Nef	A02	VLEWRFDSRL	180–189
Nef	A03/A11	QVPLRPMTYK	73–82
Nef	A03/A11	AVDLSHFLK	84–92
Nef	A11	PLRPMTYK	75–82
Nef	A24	RYPLTFGW	134–141
Nef	A33	TRYPLTFGW	133–141
Nef	B07	FPVTPQVPLR	68–77
Nef	B07	FPVTPQVPL	68–76
Nef	B07	TPQVPLRPM	71–79
Nef	B07	RPMTYKAAL	77–85
Nef	B07	TPGPGVRYPL	128–137
Nef	B07	RQDILDLWIY	106–115
Nef	B08	WPTVRERM	13–20
Nef	B08	FLKEKGGL	90–97
Nef	B1501	TQGYFPDWQNY	117–127
Nef	B1501	RMRRAEPAA	19–27
Nef	B1503	WRFDSRLAF	183–191
Nef	B18/B53	YPLTFGWCY	135–143
Nef	B2705	RRQDILDLWI	105–114
Nef	B35	VPLRPMTY	74–81
Nef	A01/A29/837/857	YFPDWQNYT	120–128
Nef	B40	KEKGGLEGL	92–100
Nef	B42	TPGPGVRYPL	128–137
Nef	B53	YPLTFGWCF	135–143
Nef	B57	HTQGYFPDWQ	116–125
Nef	B57	HTQGYFPDW	116–124
Nef	Cw07	RRQDILDLWIY	105–115
Nef	Cw7	KRQEILDLWVY	105–115
Nef	Cw8	AAVDLSHFL	83–91
Rev	A03	ERILSTYLGR	57–66
Rev	B57/B58	KAVRLIKFLY	14–23
Rev	Cw05	SAEPVPLQL	67–75
Vpu	A33	EYRKILRQR	29–37

All epitopes were referred from the Los Alamos HIV Immunology Database 2004 [24].

**Table 6:** Optimal HBV-derived HLA class I restricted CTL epitopes

Protein	HLA Restriction	Sequence	Position	Reference
Core	A2	FLPSDFFPSV	18–27	[84]
Core	A2	CLTFGRETV	107–115	[85]
Core	A2	VLEYLVSFGV	115–124	[85]
Core	A2/A24	EYLVSFGVW	117–125	[86, 87]
Core	A2	ILSTLPETTV	139–148	[86]
Core	A33/A68	STLPETTVVRR	141–151	[88]
Core	A2	AILSKTGDPV	152–161	[89]
Env	A2	LLDPRVRGL	131–139	[85]
Env	A2	VLQAGFFLL	177–185	[90]
Env	A2	FLLTRILTI	183–191	[91]
Env	A2	SLNFLGGTTV	201–210	[92]
Env	A2	FLGGTPVCL	204–212	[89]
Env	A2	LLLCLIFLL	250–258	[86]
Env	A2	LLCLIFLLV	251–259	[92]
Env	A2	LLDYQGMLPV	260–269	[92]
Env	A2	LVLLDYQGML	269–278	[85]
Env	A2	VLLDYQGML	270–278	[85]
Env	A2	LLDYQGMLPV	271–280	[85]
Env	A2	WLSLLVPFV	335–343	[92]
Env	A2	LLVPFVQWFV	338–347	[92]
Env	A2	GLSPTVWLSV	348–357	[92]
Env	A2	SIVSPFIPLL	370–379	[89]
Env	A2	LLPIFFCLWV	378–387	[92]
Env	A2	ILSPFFFLPLL	382–390	[85]
x-Protein	A2	VLCLRPVGA	15–23	[93]
x-Protein	A2	TLPSPSSSA	36–44	[93]
x-Protein	A2	HLSLRGLFV	52–60	[93]
x-Protein	A2	VLHKRTLGL	92–100	[93]
x-Protein	A2	AMSTTDLEA	102–110	[93]
x-Protein	A2	CLFKDWEEL	115–123	[93]
Pol	A24	LYSSTVPVF	62–70	[90]
Pol	A2	GLSRYVARL	455–463	[90]
Pol	A2	YMDDVVLGA	551–559	[91]
Pol	A2	FLLSLGIHL	575–583	[90]
Pol	A24	KYTSFPWLL	756–764	[87]
Pol	A2	ILRGTSFVYV	773–782	[91]
Pol	A2	SLYADSPSV	816–824	[91]

### ELISpot assay

96-well polyvinylidene plates (Millipore, Bedford, MA), pre-coated overnight with 2 μg/ml of anti-interferon gamma (IFN-γ) mAb 1-D1K (Mabtech, Stockholm, Sweden), were washed six times with sterile phosphate buffered saline (DPBS, no Ca & Mg, Mediatech) containing 1% fetal calf serum (FCS) before use. After washing, 30 μl of R10 were added to each well to avoid drying of the membrane, and 100,000 to 200,00 cells per well were added in 100 μl R10. 100,00 cells/well were used to detect responses to HIV, CMV and EBV, whereas responses to HCV and HBV were tested using 200,000 cells/well. Each peptide was added at a final concentration of 14 μg/ml (both single peptides as well as pools). As a negative control, cells were incubated in medium alone, and PHA was added at a concentration of 1.8 μg/ml to serve as a positive control. Plates were incubated for 16 h at 37°C with 5% CO_2 _before being developed. After washing six times with PBS, 100 μl of biotinylated anti-IFN-γ mAB 7-B6-1 (0.5 μg/ml, Mabtech) were added and plates were incubated for 1 hour at room temperature (RT). The plates were washed again and incubated with a 1:2000 dilution of streptavidin-coupled alkaline phosphatase (Streptavidin-ALP-PQ Mabtech) for 1 hour at RT in the dark. After washing the plates again, IFN-γ production was detected as dark spots after a short incubation of 10–20 minutes with nitroblue tetrazolium and 5-bromo-4-chloro-3-indolyl phosphate (BioRad, Hercules, CA). The color reaction was stopped by washing plates with tap water and the plates were air-dried before counting using a AID ELISPOT Reader Unit (Autoimmun Diagnostika GmbH, Strassberg, Germany). Results were expressed as spot forming cells (SFC) per million input cells. Thresholds for positive responses were determined as either 5 spots (50 SFC/10^6 ^input cells) or as the mean plus 3 standard deviations of negative control wells, whichever was higher.

### RecycleSpot

After overnight incubation in a primary ELISpot assay, cells from all wells of the ELISpot plate were transferred to a 96-well round-bottom plate and incubated at 37°C with 5% CO2 while developing the ELISpot assay. Cells from wells without any spots (including negative control wells) were then pooled, counted and used for secondary ELISpot assays. In control experiments, cells corresponding to wells with positive responses were also pooled, washed extensively (>5 times) and re-used in subsequent, secondary ELISpot assays as well. Cells from positive control wells (PHA stimulated) were not used for subsequent assays.

## Results

### Design of the optimal epitope matrix for five viral infections

To simultaneously test CTL responses against five different viruses with a limited number of PBMCs, a peptide matrix approach was used that included all previously published, well-defined CTL epitopes in HIV, HCV, HBV, EBV and CMV. The total number of described CTL epitopes for these viruses varied from 37 described optimal epitopes in HBV to more than 170 optimal epitopes in HIV. A list of all the optimal epitopes included in the present study is given in Tables 2 through 6, totaling 416 well-defined, HLA class I-restricted CTL epitopes. The included HIV epitopes were derived from the annually updated list of HIV CTL epitopes at the Los Alamos National Laboratory HIV immunology database[24]. For all the other pathogens, the epitopes listed were those for which, to the best of our knowledge, at least one publication existed showing CTL activity against this epitope in at least one infected individual. While the optimal epitopes in HIV, HCV and HBV cover large parts of their respective viral genomes, the epitopes defined in EBV and CMV represent only a portion of the proteins expressed by these viruses. Given the approximately 100 open reading frames in these large-genome viruses, complete representation of all viral proteins can hardly be achieved and most studies on these pathogens have thus focused on a relatively small number of viral proteins, especially concentrating on those containing serological determinants and those characterized by specific viral gene expression patterns. Thus, described EBV and CMV encoded CTL epitopes are derived from eleven and four different viral proteins respectively, whereas the known HIV, HCV and HBV epitopes cover all the viral proteins in these small-genome pathogens.

As the number of described optimal CTL epitopes varies between pathogens, separate peptide matrices were designed for each virus (Table 1). Importantly, the first set of pools ("protein pools") was designed so that the pools contained all the epitopes derived from the same viral protein, whereas the second half of matrix peptide pools contained the epitopes in a non-protein specific composition ("random pools"). This matrix design allowed assessment of the virus specific immune response at different levels of resolution including i) a "total virus" specific response by adding up all the protein pool specific or random peptide pool specific responses, ii) a "protein" specific responses by focusing on single pools containing all the epitopes of a given protein; and iii) upon single peptide confirmation, on a single epitope level, by comparing responses in pools containing the same epitope. Together, the epitope matrix design facilitated the assessment of T cell responses to more than 400 CTL epitopes from five different viruses simultaneously, using less than 10 × 10^6 ^PBMCs while still allowing determination of breadth and magnitude of virus-, protein-, and epitope-specific responses for each virus separately.

**Figure 1:**
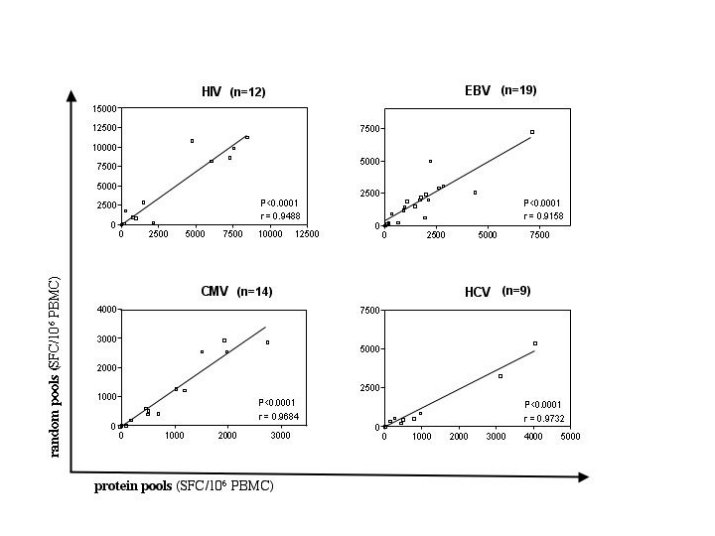
**Comparable magnitude of responses detected by "protein" and "random" peptide pools: **The magnitude of CTL responses was determined by adding magnitudes for all "protein" or "random" pools for each virus. Responses on the Y-axes represent the total of all virus specific "random pools", the X-axes indicate total responses detected using the "protein pools". Data from 12 HIV-, 19 EBV-, 14 CMV-, 9 HCV-infected individuals were tested against either set of peptide pools for A) HIV, B) HCV, C) EB V, and D) CMV and compared using the non-parametric Wilcoxon matched pairs test.

Moreover, since each epitope is tested twice in different pools, it should reflect the same magnitude of response in each pool, thus the matrix approach provides its own internal control. Additionally, "protein pools" and "random pools" should theoretically yield the same total magnitude of responses since they, as a whole, contain the same set of peptides. To test this, and rule out the possibility that peptide compositions in the different pools interfered with the detection of specific responses, the magnitude of all "protein pool" and "random pool" specific responses were compared in 19 subjects infected with EBV (n = 19) and co-infected with CMV (n = 14), HIV (12), and HCV (9). These analyses showed a statistically highly significant correlation between total magnitudes of responses detected by either set of peptide pools, indicating that the peptide mixtures in the pools sharing a specific response did not significantly impact the detection of the targeted epitope (Figure 1). Of note, for all four viruses analyzed, the "random pools" detected a slightly higher, statistically not significant total virus-specific response than the "protein pools". This is likely due to the presence of highly reactive epitopes which, when tested in the same peptide pool, can exceed the upper detection limit of the ELISpot assay and may thus underestimate the total virus-specific magnitude of responses. This may be more likely for epitopes in "peptide pools" than "random pools" if some proteins elicit generally stronger immune responses than others. A protein pool accumulating strongly reactive epitopes would result in fewer spots than the total of the respective "random pools" containing these epitopes equally distributed and fully quantitative.

### Cells from negative ELISpot wells can be used in secondary ELISpot assays (RecycleSpot)

In order to maximize cell use in samples with limited cell availability, we investigated whether cells from initial ELISpot matrix screens could be re-used in subsequent functional assays. Specifically, cells from wells that did not respond to peptides added in the first assay as well as the cells in the negative control wells may be used for secondary ELISpot assays. To assess the feasibility of this strategy, all wells from the initial ELISpot plate were transferred to a 96-well plate and incubated at 37°C with 5% CO_2 _while the ELISpot plate was developed. Cells from negative ELISpot wells were then used to confirm the identity of the epitope(s) targeted in the matrix peptide pools. In separate experiments, cells from initially positive wells were also tested in subsequent assays to determine if continuing IFN-γ production in these cells would prevent them from being used in further ELISpot assays. The analyses also compared ELISpot results in plates that were either undisturbed, or from which cells were transferred for later use.

**Figure 2:**
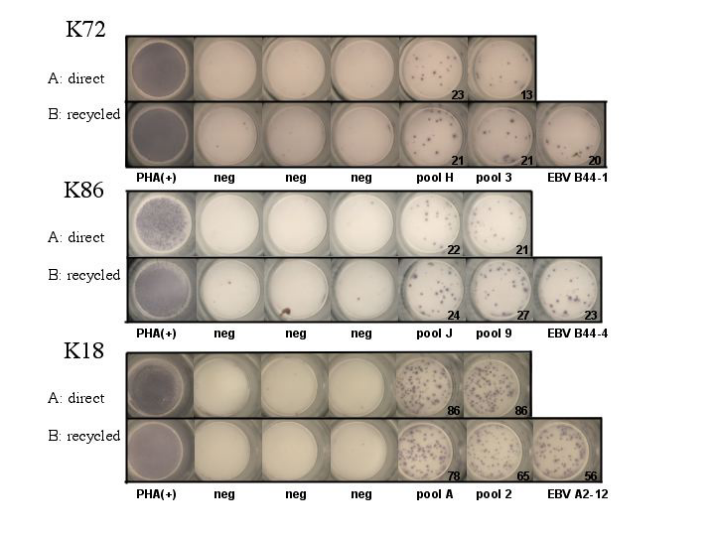
**RecycleSpot using recycled cells for the de-convolution of positive peptide pools: **Wells of primary ELISpot and secondary RecycleSpot are shown. Line A shows the data from the initial ELISpot assay, including two positive wells indicating cellular response to EBV peptide pools, three negative and one positive control wells. Line B shows the same outline as in A, this time with recycled cells in a secondary ELISpot analysis and, one separate well, using the predicted targeted epitopes from the matrix analysis. The numbers indicate the spot forming cells per million PBMC.

Representative RecyleSpot assays using PBMC and recovered cells from initial ELISpot assays from three individuals are shown in Figure 2. In all cases, negative wells from initial peptide matrix ELISpot assays were re-used to reconfirm the identity of the presumed, single targeted epitope shared by the two pools. Further, initially positive pools were re-tested to assess whether recycled cells responded with a different magnitude compared to the initial assay. The data show that sufficient cells were recovered from initial assays to perform reconfirmations of single targeted epitopes in the RecycleSpot, and that background activity and magnitude of responses were not significantly different between the first and the subsequent assays. RecycleSpot assays that used initially positive wells, or mixtures of initially positive and negative wells, showed high background in the secondary assay, indicating ongoing IFN-γ production and thus precluding these cells from use in the RecycleSpot (data not shown). No effects on the quality and the number of spots between the manipulated and non-manipulated wells were observed, indicating that harvesting cells from the ELISpot plate did not negatively interfere with the quality of the assay, at least when cells are removed by careful pipetting using a 12-channel pipetor Furthermore, RecycleSpot assays were performed using both fresh and frozen/thawed cells and showed that HIV-and EBV-specific responses were maintained in recycled cells in both cases (data not shown). Together, the data indicate that RecycleSpot can provide sufficient numbers of cells from initial assays and that these cells maintain functional capacity for use in subsequent assays, without raising background activity. Also, the data show that re-using the cells form negative wells after an overnight incubation did not reduce the magnitude of responses to a statistically significant level.

### In vitro expanded T cells mount responses detected in fresh ex vivo PBMC samples

Even though rational optimal epitope matrix design and RecycleSpots may help in reducing the required cell numbers for in vitro analyses, cell availability may still be limiting in settings where only very small biological samples can be obtained. In such instances, investigators have resorted to the use of in vitro expanded cells [19,20,25]. However, despite its potential usefulness in situations of small sample size (e.g. tissue biopsies or small volume peripheral blood samples), relatively little is known on how in vitro expansion impacts magnitude and breadth of detectable responses [20,25]. Furthermore, CTL responses to pathogens like HIV, for which a defect in their proliferative capacity has been shown, may be severely distorted by in vitro expansion, even when stimulated unspecifically [7]. To address this issue and to investigate whether stimulation of PBMC with an anti-CD3 mAb (12F6) expands CTL of different specificity equally well, we tested cells either directly or after expansion against peptide sets of described HIV- and EBV-specific epitopes restricted by the individual's HLA alleles.

**Figure 3:**
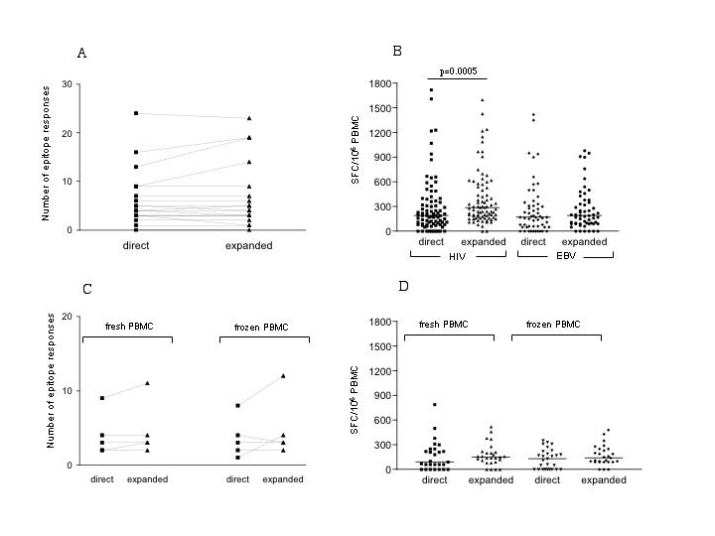
**In vitro expansion of thawed cells increases the magnitude and breadth of HIV and EBV specific responses: **Thawed PBMC from 12 individuals were tested against HIV and EBV peptide pools (n = 7 subjects) or against EBV peptide pools only (n = 5). Cells were used either directly after thawing or after thawing and a subsequent two-week in vitro expansion using the anti-CD3 mAb 12F6. A) The breadth of the detected responses (number of peptide pools reacting) and B) the total magnitude (sum of all positive peptide pools) is compared between the two cell preparation using the non-parametric Wilcoxon matched pairs test. C) PBMC from 5 EBV infected individuals were used either directly after isolation of after a two-week in vitro expansion or as frozen/thawed cells with and without in vitro expansion, and compared for the breadth (number of pools recognized) and D) total magnitude of the EBV specific responses.

These analyses included twelve subjects, of which seven were tested for responses to HIV and EBV epitopes, while the remaining five were tested for EBV-specific responses only (Figure 3).

In a first analysis, frozen PBMC were either tested directly or after a 2-week stimulation using 12F6 and the number of targeted HIV or EBV epitopes were compared, resulting in 19 data points (seven individuals tested for HIV and EBV responses and five subjects tested for EBV-specific responses). Flow cytometry in nine individuals showed preferential expansion of CD8 T cells, as CD4 expressing T cells ranged between 0.5% and 14% only, independent of HIV infection and starting CD4 T cell counts (data not shown). The Elispot results revealed no difference in the breadth of responses (number of targeted epitopes) between the directly tested and the expanded cells, as a median of 6.4 and 6.9 positive responses were detected for HIV and EBV, respectively (Figure 3A). The recognition of HIV- and EBV-derived epitopes was equally frequent by the two different cell preparations (data not shown). When the magnitude of responses was compared between directly used and expanded cells, expanded cells responded with a slightly higher magnitude than unexpanded cells. This trend was more prominent when HIV and EBV responses were analyzed separately. The HIV responses in directly tested cells showed a median of 185 SFC/10^6 ^PBMC, as compared to 285 SFC/10^6 ^PBMC in expanded cells (p = 0.0005); whereas the median EBV-specific responses had a magnitude of 170 SFC/10^6 ^in unexpanded PBMC compared to 190 SFC/10^6^PBMC in expanded cells (p > NS).

Moreover, to determine whether freshly isolated cells could also be expanded without drastic changes in their response patterns, PBMC from five EBV-infected subjects were tested directly after isolation, or after freezing, and with or without in vitro expansion. In agreement with the data from frozen samples, no significant difference in the number of pools targeted or the median magnitude of these responses was observed (Figure 3 C and 3 D). Despite concordance among the response patterns between the different cell preparations that was as low as 80%, the overall breadth and magnitude of these responses did not change. In addition, when comparing the magnitudes of the responses between each other, the relative magnitude of the responses was maintained between the four different cell preparations (data not shown). Combined, the data demonstrate that anti-CD3 expanded cells maintain their specificity and relative magnitudes when compared to unexpanded cells (both when used fresh or after thawing) indicating that in vitro expansion could be employed when the breadth, but not the absolute magnitude of responses, is being assessed. This was the case for the assessment of HIV- as well as the EBV-specific responses, suggesting that cells specific for HIV do not significantly differ from EBV specific cells in their ability to undergo in vitro expansion using a non-antigenic stimulus.

## Discussion

Cell availability can severely hamper in vitro analyses of antigen specific immune responses, hence approaches which optimize cell use are urgently needed. This is especially true for assays requiring extensive sets of antigens to be tested while only a limited number of cells can be obtained. However, logistic considerations may prevent repetitive sample collection for larger trials, and re-use of fresh or frozen samples could provide more effective ways to perform necessary analyses. The present study introduces a novel approach by which some of the sample limitations can be overcome, and may prove helpful in routine laboratory tests that currently do not make optimal use of available cells. This may not only facilitate currently performed assays, but may open possibilities to expand analyses to simultaneous assessment of even larger sets of antigens and additional functional aspects.

In the present study, we have designed and tested an approach that allows the assessment of the CTL mediated immunity against five different viral infections, including HIV, HCV, HBV, EBV and CMV. We provide an up-to-date listing of currently determined viral epitopes for which the minimal length and HLA restriction have been established. In the case of the small genome viruses HIV and HCV, these optimal epitopes represent a large portion of the respective immune targets [26]. Although they do not include all responses detected in OLP screenings, our comparative analyses of HIV-specific responses from 100 individuals detected by either overlapping peptide (OLP) sets or optimal epitopes show that on average 68% of the observed OLP responses are covered by previously established HIV optimal epitopes (data not shown).

The present data also show that PBMC recycled from negative wells from an ELISpot assay can be re-used for subsequent functional assays. Depending on the analyses performed in the subsequent assay, such as reconfirmation of single epitope responses predicted in the initial matrix analyses, relatively small numbers of cells maybe required. Thus, although individuals with broad responses in the initial ELISpot assay will not yield many negative wells from which to recycle cells, the wells with non-targeted peptide in addition to the negative control wells often provide sufficient quantities of recycled cells to complete the matrix based analyses. Since the responses in the RecycleSpot are not significantly diminished as compared to the initial assay (Figure 2), the magnitude of responses in the subsequent assay can still provide adequate data at the single epitope level.

In vitro expanded cells have been used in a number of studies where cell availability has been the limiting factor[21,22]. However, no study has directly compared for instance biopsy and PBMC-derived responses in a systematic manner and on a single epitope level, and it is unclear whether the in vitro expansion provides identical data. In the present report, we have compared the response patterns to EBV- and HIV-derived antigens in directly ex vivo and in vitro expanded PBMC preparations. No significant differences were observed, although some responses are lost or gained upon expansion. As no difference in the concordance between EBV- and HIV-specific responses was observed, the data indicate that responses to both viruses are equally well expandable in vitro using an antigen-unspecific stimulus, despite the ongoing viral replication in most HIV infected subjects tested here.

Thus, optimal epitope matrices, RecycleSpot and in vitro expansion of cells can be combined to achieve maximal information on an extensive set of antigens, even if sample availability is limited. As a practical approach, expanded cells from frozen PBMC aliquots can be used initially to screen a large number of antigens to determine the approximate breadth of responses within the set of antigens used. Subsequent studies using unexpanded cells and antigen matrices in conjunction with RecycleSpot would then allow determination of the true breadth and, more importantly, the true magnitude of these responses while requiring minimal cell numbers. Furthermore, cells can be successfully recovered from the RecycleSpot once more to be used for genetic analyses such as HLA typing. This combined approach should facilitate future work in settings in which cell availability is of constant concern.

**Table 3:** Optimal EBV-derived HLA class I restricted CTL epitopes

Protein	HLA Restriction	Sequence	Position	Reference
BMLF1	A1	LVSDYCNVLNKEFT	25–39	[27]
BMLF1	A2	GLCTLVAML	280–288	[28]
BMLF1	B18	DEVEFLGHY	397–405	[28]
BMLF1	n.d.*	KDTWLDARM	265–273	[29]
BMLF1	A24	DYNFVKQLF	320–328	[30]
BHRF	A2	LLWAARPRL	204–212	[31]
BZLF1	B7	LPCVLWPVL	44–52	[13]
BZLF1	B8	RAKFQLL	190–197	[32]
BZLF1	Cw6	RKCCRAKFKQLLQH	186–201	[1]
BMRF1	Cw6	YRSGIIAW	268–276	[16]
BMRF1	Cw3	FRNLAYGRTCVLGK	86–100	[16]
BRLF1	A2	YVLDHLIVV	109–117	[33]
BRLF1	A2	RALIKTLPRASYSSH	225–239	[27]
BRLF1	A3	RVRAYTYSK	148–156	[1]
BRLF1	A11	ATIGTAMYK	134–142	[16]
BRLF1	A24	DYCNVLNKEF	28–37	[27]
BRLF1	A24	TYPVLEEMF	198–206	[30]
BRLF1	B61	QKEEAAICGQMDLS	529–543	[1]
BRLF1	Cw4	ERPIFPHPSKPTFLP	393–407	[1]
gp110	A2	ILIYNGWYA	106–114	[1]
gp110	B35	VPGSETMCY	544–552	[1]
gp110	B35	APGWLIWTY	190–198	[1]
gp85	A2	TLFIGSHVV	420–428	[1]
gp85	A2	LMPIIPLINV	542–550	[1]
gp85	A2	SLVIVTTFV	225–233	[1]
gp350	A2	VLQWASLAV	863–871	[1]
gp350	A2	VLTLLLLLV	871–879	[34]
gp350	A2	LIPETVPYI	152–160	[34]
gp350	A2	QLTPHTKAV	67–75	[34]
EBNA1	A2	FMVFLQTHI	562–570	[13]
EBNA1	B7	RPQKRPSCI	72–80	[35]
EBNA1	B7	IPQCRLTPL	528–536	[35]
EBNA1	B53	HPVGEADYF	407–415	[35]
EBNA2	A2/B51	DTPLIPLTIF	42–50	[36]
EBNA3A	A2	SVRDRLARL	596–604	[37]
EBNA3A	A3	RLRAEAQVK	603–611	[38]
EBNA3A	A24	RYSIFFDY	246–253	[37]
EBNA3A	A29	VFSDGRVAC	491–499	[16]
EBNA3A	A30	AYSSWMYSY	176–184	[1]
EBNA3A	B7	RPPIFIRRL	379–387	[39]
EBNA3A	B7	VPAPAGPIV	502–510	[16]
EBNA3A	B8	QAKWRLQTL	158–166	[37]
EBNA3A	B8	FLRGRAYGL	325–333	[40]
EBNA3A	B35	YPLHEQYGM	458–466	[37]
EBNA3A	B46	VQPPQLTLQV	617–625	[41]
EBNA3A	B62	LEKARGSTY	406–414	[16]
EBNA3A	n.d.*	HLAAQGMAY	318–326	[16]
EBNA3B	A1l	NPTQAPVIQLHAVY	101–115	[40]
EBNA3B	A1l	AVFDRKSDAK	399–408	[16]
EBNA3B	A1l	LPGPQVTAVLLHEES	481–495	[40]
EBNA3B	A1l	DEPASTEPVHDQLL	551–563	[40]
EBNA3B	Al1	IVTDFSVIK	416–424	[40]
EBNA3B	A24	TYSAGIVQI	217–225	[16]
EBNA3B	A27	RRARSLSAERY	243–253	[42]
EBNA3B	B35	AVLLHEESM	488–496	[1]
EBNA3B	B44	VEITPYKPTW	657–666	[16]
EBNA3B	B58	VSFIEFVGW	279–287	[43]
EBNA3B	B62	GQGGSPTAM	831–839	[16]
EBNA3C	B7	QPRAPIRPI	881–889	[39]
EBNA3C	B27	RRIYDLIEL	258–266	[44]
EBNA3C	B27	HRCQAIRK	149–157	[16]
EBNA3C	B27	FRKAQIQGL	343–351	[16]
EBNA3C	B27	RKIYDLIEL	258–266	[45]
EBNA3C	B27	RRIFDLIEL	258–266	[45]
EBNA3C	B27	LRGKWQRRYR	249–258	[44]
EBNA3C	B37	LDFVRFMGV	285–293	[46]
EBNA3C	B39	HHIWQNLL	271–278	[16]
EBNA3C	B44	KEHVIQNAF	335–343	[47]
EBNA3C	B44	EENLLDFVRF	281–290	[40]
EBNA3C	B44	EGGVGWRHW	163–171	[48]
EBNA3C	B62	QNGALAINTF	213–222	[49]
EBNALP	A2	SLREWLLRI	284–292	[43]
LMP1	A2	YLQQNWWTL	159–167	[50]
LMP1	A2	YLLEMLWRL	125–133	[50]
LMP1	A2	LLVDLLWLL	167–175	[50]
LMP1	A2	TLLVDLLWL	166–174	[50]
LMP1	A2	LLLIALWNL	92–100	[50]
LMP1	B51	DPHGPVQLSYYD	393–404	[51]
LMP2	A2	FLYALALLL	356–364	[52]
LMP2	A2	LLWTLWLL	329–337	[53]
LMP2	A2	CLGGLLTMV	426–434	[54]
LMP2	A2	LTAGFLIFL	453–461	[53]
LMP2	A11	SSCSSCPLSKI	340–350	[53]
LMP2	A23	PYLFWLAAI	131–139	[55]
LMP2	A2	LLSAWILTA	447–455	[43]
LMP2	A24	TYGPVFMCL	419–427	[53]
LMP2	A24	IYVLVMLVL	222–230	[30]
LMP2	A25	VMNSNTLLSAW	442–451	[16]
LMP2	A27	RRRWRRLTV	236–244	[44]
LMP2	B40	IEDPPFNSL	200–208	[53]
LMP2	B63	WTLWLLI	331–338	[1]

*not determined

**Table 4:** Optimal CMV-derived HLA class I restricted CTL epitopes

Protein	HLA Restriction	Sequence	Position	Reference
pp65	B35	IPSINVHHY	123–131	[56]
pp65	B35	DDVWTSGSDSDEELV	397–411	[57]
pp65	B35	VFPTKDVAL	187–195	[17]
pp65	B38	PTFTSQYRIQGKL	367–379	[17]
pp65	B7	TPRVTGGGAM	417–426	[57]
pp65	B7	RPHERNGFTVL	265–275	[17]
pp65	A1	YSEHPTFTSQY	363–373	[17]
pp65	A1101	SVLGPISGHVLK	13–24	[17]
pp65	A2402	FTSQYRIQGKL	369–379	[17]
pp65	A68	FVFPTKDVALP	186–196	[17]
pp65	A2	NLVPMVATV	495–503	[57]
pp65	A2	VLGPISGHV	14–22	[58]
pp65	A2	MLNIPSINV	120–128	[58]
pp65	B44	EFFWDANDIY	512–521	[57]
pp65	A2402	VYALPLKML	113–121	[59]
pp65	A2402/Cw0401	QYDPVAALF	341–349	[60, 61]
pp65	B5201	QMWQARLTV	155–163	[62]
pp65	A0207	RIFAELEGV	522–530	[61]
pp65	A1101	ATVQGQNLK	501–509	[61]
pp65	B1501	KMQVIGDQY	215–223	[61]
pp65	B4001	CEDVPSGKL	232–240	[61]
pp65	B40	HERNGFTVL	267–275	[61]
pp65	B4006	AELEGVWQPA	525–534	[61]
pp65	B4403	SEHPTFTSQY	364–373	[61]
pp65	B5101	DALPGPCI	545–552	[61]
pp65	Cw0102	RCPEMISVL	7–15	[61]
pp65	Cw0801	VVCAHELVC	198–206	[61]
pp65	Cw1202	VAFTSHEHF	294–302	[61]
pp65	A33	SVNVHNPTGR	91–100	[63]
pp150	A0301	TTVYPPSSTAK	945–955	[17]
pp150	A68	QTVTSTPVQGR	792–802	[17]
IE	B7	CRVLCCYVL	309–317	[64]
IE	A2	YILEETSVM	315–323	[65]
IE	B18	ELKRKMIYM	199–207	[65]
IE	B18	CVETMCNEY	279–287	[65]
IE	B18	DEEDAIVAY	379–387	[65]
IE	B18	SDEEEAIVAYTL	378–389	[56]
GB	A2	FIAGNSAYEYV	618–628	[66]

**Table 5:** Optimal HCV-derived HLA class I restricted CTL epitopes

Protein	HLA Restriction	Sequence	Position	Reference
Core	B60	GQIVGGVYLL	28–37	[67]
Core	A0201	YLLPRRGPRL	35–44	[68]
Core	B7	GPRLGVRAT	41–49	[69]
Core	B44	NEGCGWAGW	88–96	[70]
Core	A0201	DLMGYIPLV	132–140	[71]
Core	A0201	ALAHGVRAL	150–158	[15]
Core	A0201	LLALLSCLTV	178–187	[72]
Core	A11	MSTNPKPQK	1–9	[73]
P7	A29	FYGMWPLLL	790–798	[15]
P7	Cw7	FYGMWPLL	790–797	[15]
E1	A0201	ILHTPGCV	220–227	[74]
E1	B35	NASRCWVAM	234–242	[25]
E1	A0201	QLRRHIDLLV	257–266	[74]
E1	A23	FLVGQLFTF	285–293	[15]
E1	A0201	MMMNWSPTT	322–330	[15]
E1	A0201	SMVGNWAKV	363–371	[74]
E1	B35	CPNSSIVY	207–214	[15]
E2	A0201	SLLAPGAKQNV	401–411	[74]
E2	B53	CRPLTDFDQGW	460–469	[69]
E2	B51	YPPKPCGI	489–496	[73]
E2	B60	GENDTDVFVL	530–539	[75]
E2	B50	CVIGGAGNNT	569–578	[73]
E2	A0201	RLWHYPCTV	614–622	[76]
E2	A11	TINYTIFK	621–628	[69]
E2	B60	LEDRDRSEL	654–662	[75]
E2	A2402	EYVLLLFLL	717–725	[77]
E2	B57	NTRPPLGNWF	541–550	[15]
NS2	A29	MALTLSPY	827–834	[25]
NS2	A25	SPYYKRYISW	832–841	[78]
NS2	A23	YISWCLWWL	838–845	[69]
NS3	A24	AYSQQTRGL	1031–1039	[79]
NS3	A0201	CINGVCWTV	1073–1081	[68]
NS3	A0201	LLCPAGHAV	1169–1177	[68]
NS3	A0201	LLCPSGHAV	1169–1177	[68]
NS3	A11	TLGFGAYMSK	1261–1270	[80]
NS3	A0201	ATLGFGAYM	1260–1268	[81]
NS3	A0201	TLHGPTPLL	1617–1625	[81]
NS3	A0201	TGAPVTYSTY	1287–1296	[79]
NS3	A2402	TYSTYGKFL	1292–1300	[77]
NS3	B35	HPNIEEVAL	1359–1367	[82]
NS3	B8	HSKKKCDEL	1395–1403	[69]
NS3	A0201	KLVALGINAV	1406–1415	[68]
NS3	B8	LIRLKPTL	1611–1618	[75]
NS3	A11	TLTHPVTK	1636–1643	[80]
NS3	A68	HAVGLFRAA	1175–1184	[15]
NS3	A0201	GLLGCIITSL	1038–1047	[81]
NS4	A2402	FWAKHMWNF	1760–1768	[77]
NS4	B35	IPDREVLY	1695–1712	[15]
NS4	A24	VIAPAVQTNW	1745–1754	[15]
NS4	B57	LTTSQTLLF	1801–1809	[15]
NS4B	A25	EVIAPAVQTNW	1744–1754	[78]
NS4B	A25	ETFWAKHMW	1758–1766	[78]
NS4B	A0201	SLMAFTAAV	1789–1797	[68]
NS4B	A0201	LLFNILGGWV	1807–1816	[72]
NS4B	A0201	ILAGYGAGV	1851–1859	[72]
NS4B	B37	SECTTPCSGSW	1966–1976	[78]
NS4B	B38	AARVTAIL	1941–1948	[75]
NS5	A2	VLSDFKTWL	1987–1995	[83]
NS5	B35	EPEPDVAVL	2162–2170	[15]
NS5	B57	LGVPPLRAWR	2912–2921	[15]
NS5A	B60	HEYPVGSQL	2152–2160	[75]
NS5A	B35	PCEPEPDVAVL	2161–2171	[75]
NS5A	B38	NHDSPDAEL	2218–2226	[75]
NS5A	A2	SPDAELIEANL	2221–2231	[75]
NS5A	A25	ELIEANLLW	2225–2233	[78]
NS5A	A0201	ILDSFDPLV	2252–2260	[68]
NS5A	B60	REISVPAEIL	2267–2275	[80]
NS5B	A3	SLTPPHSAK	2510–2518	[80]
NS5B	A3	RVCEKMALY	2588–2596	[69]
NS5B	A2	ALYDWTKL	2594–2602	[78]
NS5B	B57	KSKKTPMGF	2629–2637	[80]
NS5B	A0201	GLQDCTMLV	2727–2735	[72]
NS5B	B38	HDGAGKRVYL	2794–2804	[80]
NS5B	A25	TARHTPVNSW	2819–2828	[78]
NS5B	A2402	RMILMTHFF	2841–2849	[77]
NS5B	A2402	CYSIEPLDL	2870–2878	[77]
NS5B	A31	VGIYLLPNR	3003–3011	[80]
